# What’s old is new again: yeast mutant screens in the era of pooled segregant analysis by genome sequencing

**DOI:** 10.15698/mic2016.04.488

**Published:** 2016-04-04

**Authors:** Chris Curtin, Toni Cordente

**Affiliations:** 1 The Australian Wine Research Institute, Urrbrae, SA 5064, Australia.

**Keywords:** QTL, genetics, polygenic analysis, volatile aroma compound, flavour

## Abstract

While once *de-rigueur* for identification of genes involved in biological processes, screening of chemically induced mutant populations is an approach that has largely been superseded for model organisms such as *Saccharomyces cerevisiae*. Availability of single gene deletion/overexpression libraries and combinatorial synthetic genetic arrays provide yeast researchers more structured ways to probe genetic networks. Furthermore, in the age of inexpensive DNA sequencing, methodologies such as mapping of quantitative trait loci (QTL) by pooled segregant analysis and genome-wide association enable the identification of multiple naturally occurring allelic variants that contribute to polygenic phenotypes of interest. This is, however, contingent on the capacity to screen large numbers of individuals and existence of sufficient natural phenotypic variation within the available population. The latter cannot be guaranteed and non-selectable, industrially relevant phenotypes, such as production of volatile aroma compounds, pose severe limitations on the use of modern genetic techniques due to expensive and time-consuming downstream analyses. An interesting approach to overcome these issues can be found in Den Abt *et al*. 1 (this issue of *Microbial Cell*), where a combination of repeated rounds of chemical mutagenesis and pooled segregant analysis by whole genome sequencing was applied to identify genes involved in ethyl acetate formation, demonstrating a new path for industrial yeast strain development and bringing classical mutant screens into the 21^st^ century

Fermented foods and beverages, such as beer, wine, saké, and bread, owe much to the primary fermentation yeast used in their production*, Saccharomyces cerevisiae*. In addition to the crucial role it plays in conversion of sugars to ethanol and carbon dioxide, *S. cerevisiae* is responsible for an important fraction of fermented product flavour and aroma through biosynthesis of esters, higher alcohols, volatile fatty acids, and low-molecular weight sulfur compounds 2. Biochemical pathways have been established for most compounds within these families, a particularly well-studied example being the Ehrlich higher-alcohol pathway 3. Nevertheless, the genetic determinants of metabolic processes for production of some compounds remain elusive. One of the most significant esters produced by yeast is ethyl acetate, which imparts a "fruity", "confectionary" aroma at low concentrations, but at high concentrations is considered an off-flavour with a "solvent"-like aroma. Two alcohol acetyltransferase-encoding genes, *ATF1* and *ATF2*, are responsible for production of the related ester, 3-methylbutyl acetate, yet when both genes are deleted approximately 50% of ethyl acetate production remains 4, highlighting the polygenic nature of this important metabolic trait.

Pooled segregant analysis is an ideal tool for the study of polygenic traits in *S. cerevisiae*, yet has seen only sporadic application to production of volatile aroma compounds 5,6,7. Costly chemical analyses and the requirement to perform micro-fermentations of sufficient scale has undoubtedly presented a barrier for many researchers considering taking this approach, particularly because of the need for up-front large-scale screens of *S. cerevisiae* strains for production of each target metabolite 8, to identify those with significant differences. The work of Den Abt *et al*. 1 shows that a chemically mutagenised parent strain represents a more efficient way into the process, obviating the need for large collections of "natural" *S. cerevisiae* strains as the source of genetic variation. Indeed, in some strain collections the range of observed variation can be quite low, a case in point being extensive redundancy and genetic similarity of commercial and industry-isolated wine yeasts 9. Important to note, and a potential limitation or advantage depending upon your perspective, is that the mutant S288c variants TDA1(4) and TDA3(4) generated by Den Abt *et al*. 1 exhibited a large number of mutations not observed "naturally" across approximately 40 previously sequenced *S. cerevisiae* strains.

In the work of Den Abt *et al*. 1, a relatively a large number of mutations were generated in the haploid S288c background through performing repeat rounds of chemical mutagenesis, while monitoring a range of classical phenotypes to estimate the efficacy of their treatments and ensure retention of mating proficiency. Enough mutations, though, to reveal previously unknown genetic determinants of a complex polygenic trait?.

Pooled segregant analysis of a cross between mutant TDA1(4), obtained after 4 rounds of mutagenesis from a laboratory strain, and a high-producer of ethyl acetate, ER7A (haploid strain derived from a commercial strain), enabled the authors to identify three novel potential causative genes: *PMA1, CEM1, *and* TPS1*. The first two were identified as causative mutant alleles for lowering ethyl acetate production, while *TPS1* is a causative genetic background allele found in S288c and not ER7A.

Likely pleiotropic effects of *PMA1*, which encodes a H+-ATPase essential for maintenance of the plasma membrane proton gradient, make it difficult to interpret the role of this gene in ethyl acetate production. On the other hand, *CEM1,* a mitochondrial β-keto-acyl synthase, is a homolog of *FAS2*, which encodes for the α-subunit of cytoplasmic fatty acid synthase. A gain-of-function mutation in *FAS2 *has previously been shown to have a profound effect on the production of medium chain fatty ethyl esters, while the concentrations of 3-methylbutyl acetate and acetic acid were reduced 10,11. It has also been described that null mutations in *CEM1* result in a decreased number of lipid droplets in the cell 12, and the main enzyme involved in acetate ester production during fermentation, Atf1p, is located in lipid particles. Further characterisation of *CEM1* and its role in ethyl acetate production may in turn reveal novel links with production of other important flavour-active esters.

Coming back to an earlier question. Is it a limitation or advantage that the observed mutations in TDA1(4) and TDA3(4) after repeated rounds of mutagenesis were outside those found "naturally" amongst sequenced strains of *S. cerevisiae*? The ability to pick up both mutant and background alleles in one experiment highlights the potential power of this novel approach. Furthermore, if the goal is to identify genes that contribute to a phenotype, or to find novel alleles that confer an industrially-relevant phenotype, an efficient path to the desired endpoint is arguably more important than being able to draw conclusions about drivers of microevolution within the species. A limitation of the study is the use of a single laboratory strain genetic background to investigate a phenotype of industrial relevance in the beverage industry. The authors’ state in their conclusion that small collections of independently mutagenised strains could be established, and it is important that industrial strains are amongst them so as to enable the study of industrial performance traits that laboratory yeasts do not possess. This will undoubtedly happen in the near future and by proving its utility for the study of complex polygenic traits, Den Abt *et al*. 1 have given the humble mutant screen a new lease on life.
